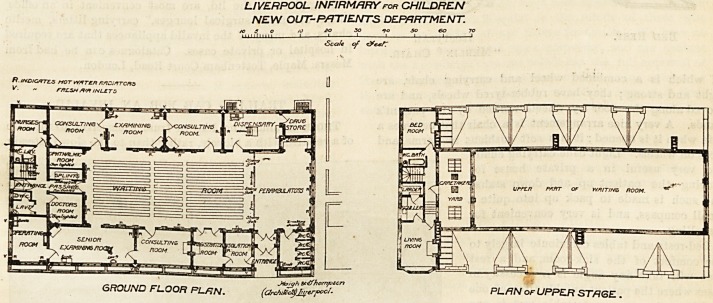# The Liverpool Infirmary for Sick Children

**Published:** 1902-10-04

**Authors:** 


### THE LIVERPOOL INFIRMARY FOR SICK CHILDREN.

This infirmary was founded in 1851, and it was the first provincial hospital devoted exclusively to children. Since being founded it has passed through many changes. During the first year about 700 patients were treated in it, and year by year this number has increased until the present time, when about 14,000 new cases are treated annually, involving between 50,000 and 60,000 attendances.

The building first used was a small private house in Upper Hill Street, and in three years a move was made to a larger house in Great George Street. This, too, soon proved inadequate, and in 1856 a house in Hope Street was rented. Before this only out-patients could be treated, but now it was possible to provide a few beds for in-patients. As generally happens in these cases the Charity expanded, and in the year 1866 the present site was obtained. The foundation-stone was laid by the Duke of Edinburgh on June 23rd of the same year. It is this building which is to be superseded by an entirely new erection. A suitable site has been obtained opposite the present one, and on this the out-patients' department is being built. By this arrangement the out- and in-patients' departments will be kept entirely distinct. The governors do not intend stopping at this point. The old infirmary is to be pulled down preparatory to the erection of a new one entirely for the in-patients. The scheme is therefore complete.

The new out-patients' department has the great advantages of being surrounded on all sides by open spaces, and it will thus possess light and ventilation on all its aspects. As the building is practically only one story high these essential conditions can be obtained with the greatest ease. The patients' entrance is at the south-west angle and is approached from Mulberry Street, being, in fact, exactly opposite the present infirmary. Near the entrance is the registration room, adjoining which is an isolation ward, by which means an infectious case can be removed without re-entering the main building. The dispensary is placed opposite the entrance.

The medical staff have an entrance in Mulberry Place. The centre of the building is, of course, taken up by the waiting room, and around it are the examining rooms, consultation rooms, operation room, ophthalmic room, and medical officer's room. The disposition of these various adjuncts seems to have been very carefully thought out, and ought to ensure harmonious working.

Over the extreme part of the north end are rooms for the caretaker. The rooms will be warmed by hot water, provision having been made for permitting fresh air to flow over the radiators. Leather's valves are also used, and all the windows have casements made to open. Roof ventilators will also be used. The interior walls will be lined with a dado of glazed bricks, and the corners and angles have been rounded off. Above the dado line the walls will be rendered in Portland cement and finished with Parian, subsequently rendered impervious with Ripolin paint. The floors are of terrazzo, which forms an admirable surface *provided it can be kept from cracking*. The admirable plan of bracketting the lavatories and sinks from the walls has been adopted. No floor supports are used, and cleaning is made easier while the chance of impurities lodging are lessened. Electric light will be used.

The architects are Messrs. Haigh and Thompson. The cost of the building is not stated.

## Figures and Tables

**Figure f1:**